# Neonatal exposure to sevoflurane may not cause learning and memory deficits and behavioral abnormality in the childhood of Cynomolgus monkeys

**DOI:** 10.1038/srep11145

**Published:** 2015-06-05

**Authors:** Lisheng Zhou, Zhi Wang, Hui Zhou, Ting Liu, Fudin Lu, Shouping Wang, Jing Li, Shuling Peng, Zhiyi Zuo

**Affiliations:** 1 Department of Anesthesiology, Sun Yat-Sen Memorial Hospital, Sun Yat-Sen University, Guangzhou, Guangdong, China; 2 Department of Obstetrics and Gynecology, Sun Yat-Sen Memorial Hospital, Sun Yat-Sen University, Guangzhou, Guangdong, China; 3 Department of Anesthesiology, University of Virginia, Charlottesville, Virginia, U.S.A

## Abstract

Results of animal studies have raised a significant concern that commonly used general anesthetics may induce neurotoxicity in children. It may be difficult to resolve this concern with human studies because randomizing children only for testing anesthetic toxicity may not be feasible. We randomized 6-day old male Cynomolgus monkeys to receive or not to receive sevoflurane anesthesia at surgical plane for 5 h. Sevoflurane is the most commonly used general anesthetic in children in the U.S.A. Here, we showed that sevoflurane anesthesia did not affect the behavior evaluated by holding cage method when the monkeys were 3 and 7 months old. However, there was an age-dependent decrease in the frequency of stress events and environmental exploration behavior during the test. Sevoflurane also did not affect the learning and memory of the monkeys when they were assessed from the age of 7 months. Finally, sevoflurane did not affect the expression of multiple neuron-specific proteins in the hippocampus and cerebral cortex of 10-month old monkeys after all behavioral and cognitive tests were completed. These results suggest that exposure of neonatal monkey to sevoflurane may not affect cognition, behavior and neuronal structures in childhood, indicating the safety of sevoflurane anesthesia in children.

Human fetal alcohol syndrome is well defined and is caused by repeated exposure of fetus to alcohol. Mental defect is an important feature of fetal alcohol syndrome^1^. Subsequent to the finding that alcohol can induce apoptosis in neonatal rat brain^2^, exposure of neonatal rodents to general anesthetics for a prolonged duration has been found to cause apoptosis of brain cells and learning and memory deficits^3^,^4^. This finding causes a significant concern in the scientific community and public, considering that about 6 million children under 15 years old and 1.5 million infants each year in the U.S.A. are exposed to anesthesia during their surgery^3^,^5^. The numbers of surgery and anesthesia exposure in kids in the world may be at least 10 times the numbers in the U.S.A. based on the estimates of total numbers of surgery performed for all age groups each year in the world and U.S.A.^6^,^7^. Many retrospective studies have been performed. It appears that children may develop cognitive and behavioral impairment if they were exposed to more than one surgery and anesthesia before the age of 4 years^3^,^8^,^9^. Since these children also often had surgery, it is not known whether the effects on cognition and behavior are due to surgery, anesthesia or the combination of surgery and anesthesia.

Since it may be very difficult to determine whether anesthetics cause brain cell death and mental defects by human studies because randomizing children to receive general anesthesia for non-medical reasons is not feasible, studies with non-human primates may provide the most useful information to answer the important question of what may happen in humans. Studies have shown that exposure of 120-day old fetuses or 6-day old newborns of monkeys to isoflurane, propofol or ketamine for 5 h induces brain cell injury/death assessed a few hours after the exposure^10^,^11^,^12^,^13^. Exposure of 5- to 6-day old monkeys to ketamine infusion for 24 h also induces long-lasting cognitive impairment^14^. However, it is not known the long-term effects of commonly used general anesthetics, especially under clinically relevant conditions. Of note, isoflurane is a volatile anesthetic. It is often used as an agent to maintain general anesthesia. It is a full general anesthetic that can induce unconsciousness, analgesia and immobility^15^. Propofol and ketamine are intravenous anesthetics. Propofol is commonly used to induce unconsciousness but do not have significant analgesic effect^16^. Ketamine has significant analgesic effect but can cause mental and mood changes including hallucination^17^. While volatile anesthetics may have multiple targets in the brain for its anesthetic properties, propofol and ketamine may work on enhancing γ-aminobutyric acid receptors or inhibiting N-methyl-D-aspartate receptors, respectively, for their effects^15^,^16^,^17^.

Sevoflurane is the most commonly used general anesthetic in pediatric patients. It has less airway stimulation than isoflurane^18^. This feature, along with other characteristics, such as lower blood solubility, has made sevoflurane a more popular volatile anesthetic than the older drug isoflurane in clinical practice^19^. Exposure of 6-day old mice to sevoflurane for 3 days, 2 h each day, induced brain cell death and learning and memory impairment. This effect did not appear if they were exposed to sevoflurane for 2 h for only one day^20^. Single exposure to sevoflurane for 6 h has been shown to cause brain cell death and impairment of learning and memory in rodents^21^,^22^, although no effects after a long exposure to sevoflurane have also been reported^23^. However, the effects of sevoflurane on neonates of animal species that are closer to humans are not reported. Thus, we designed this study to determine whether exposure of neonatal monkeys to sevoflurane at surgical plane of anesthesia induced cognitive and behavioral impairments.

## Results

All twelve animals were born full term. Half of the animals were randomized into control group and the other half were in the sevoflurane group. All animals in the sevoflurane group completed sevoflurane anesthesia at surgical plane for 5 h. The end-tidal sevoflurane concentration was 2 to 2.6% during anesthesia (Table 1). This concentration range is consistent with previous studies showing that one minimum alveolar concentration of sevoflurane in Cynomolgus monkeys is 2%^24^,^25^. The sevoflurane exposure occurred when they were 6 days old. Some animals had mild CO_2_ accumulation and acidosis during anesthesia (Table 1). The degree of these changes was within the ranges of changes in humans during anesthesia. No animals had an episode of hypoxia or hypoglycemia during sevoflurane exposure (Table 1). However, one animal in the sevoflurane group died accidently at about 4 months of age. Thus, the behavioral and cognitive data of 6 control animals and 5 animals from sevoflurane group were analyzed and presented here. Behavior was observed at the age of 3 and 7 months, respectively, and cognition test was started at the age of 7 months. Their brain was harvested when they were 10 months old after the cognitive tests were completed. One animal from each group was perfused systemically with normal saline and then 4% formaldehyde to preserve brain tissues for possible histology examination. These brain tissues were not suitable for Western blotting. Thus, Western blotting was performed with the tissues from 5 control animals and 4 animals in the sevoflurane group.

The body weights of control animals (n = 6) and animals in the sevoflurane group (n = 5) were, respectively, 367 ± 51 g and 342 ± 47 g at birth, 1237 ± 145 g and 1178 ± 123 g at the age of 7 months, and 1823 ± 154 g and 1802 ± 136 g at the age of 10 months when their brain tissues were harvested. All P values for the comparisons between control and sevoflurane groups were >0.05.

### Age but not a prior sevoflurane exposure affected the behavior of monkeys in holding cage test

Placing young un-weaned monkeys alone in a cage induces stress to them. Their environmental exploration, stress activity, and time spent in locomotion and hanging in this new but potential stressful environment were determined using the definitions published before^26^,^27^,^28^. Hanging was considered an escaping/stress response^27^.

**Figure 1:**
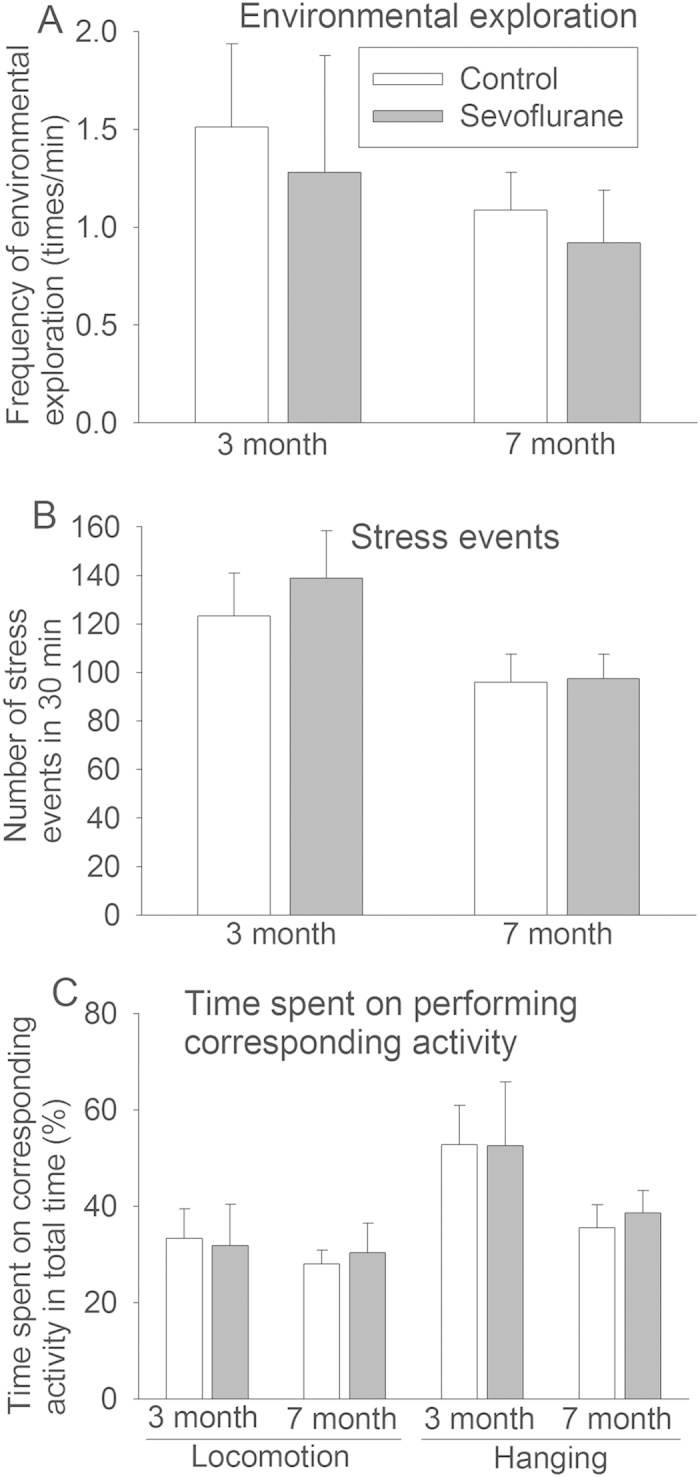
Behavior after sevoflurane exposure. Six-day old male Cynomolgus monkeys were exposed to sevoflurane anesthesia for 5 h. Holding cage test was performed when they were 3 and 7 months old. **A**: Frequency of environmental exploration activity. **B**: Frequency of stress events. **C**: Time spent on performing locomotion and hanging. Results are mean ± S.D. (n = 5 – 6).

There was no difference in the frequency of environmental exploration, stress events, and time spent in locomotion and hanging between the animals in the control and sevoflurane groups by t-test (Fig. 1). However, age is a significant factor to determine the frequency of environmental exploration (F = 5.355, P = 0.033), frequency of stress events (F = 27.786, P < 0.001), and the time in hanging (F = 19.56, P < 0.001) by two-way analysis of variance with age and sevoflurane exposure as the two factors in the analysis. Sevoflurane exposure was not a significant factor to determine those activities by this analysis (all P values > 0.05). Age or sevoflurane exposure was not a significant factor to affect the amount of time spent in locomotion. There was no significant interaction between age and sevoflurane exposure in any of the behavioral parameters.

### Sevoflurane exposure did not affect learning and memory

**Figure 2:**
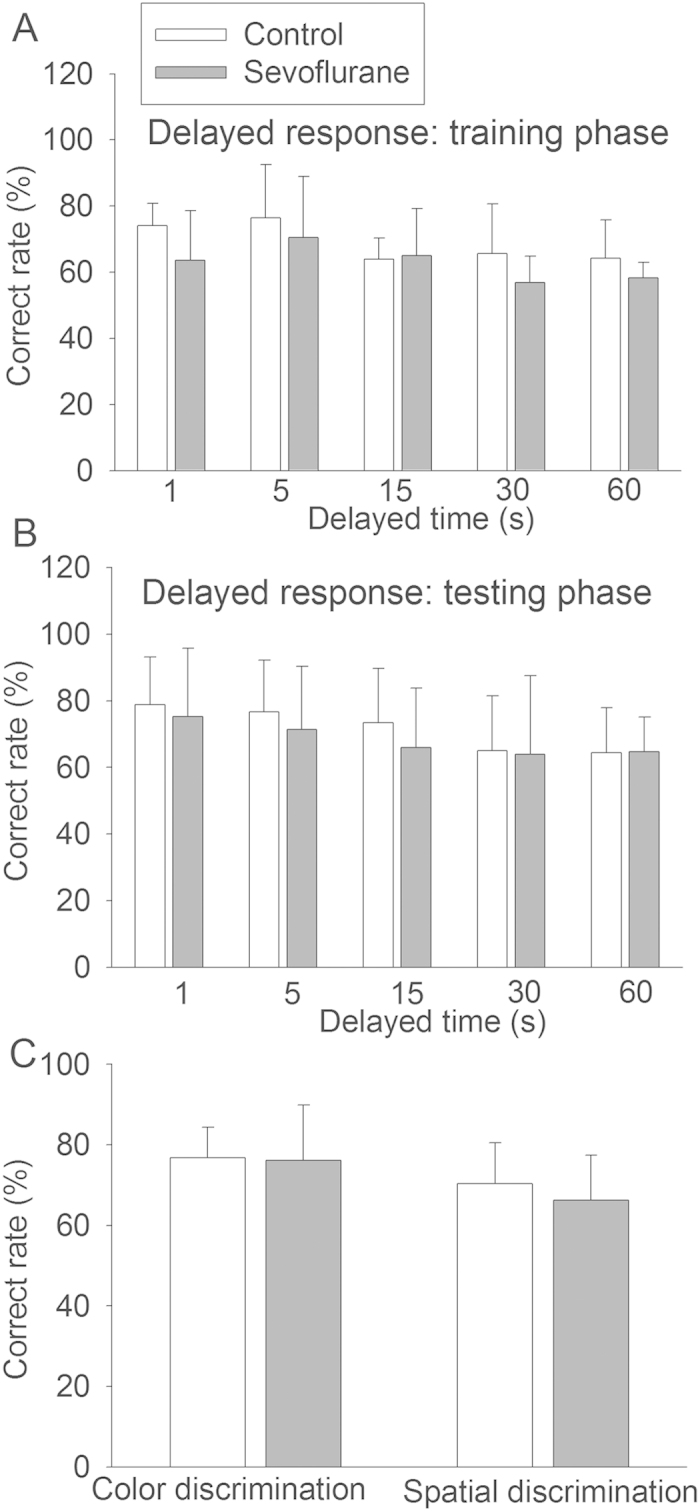
Learning and memory after sevoflurane exposure. Six-day old male Cynomolgus monkeys were exposed to sevoflurane anesthesia for 5 h. Learning and memory were started to be tested at 7 months after sevoflurane exposure. **A**: Rate of correct choice in the training phase of delayed response task. **B**: Rate of correct choice in the testing phase of delayed response task. **C**: Rate of correct choice in the color discrimination and spatial discrimination. Results are mean ± S.D. (n = 5 – 6).

We started to test the monkeys at the age of 7 months after they were weaned and old enough for reliable responses in learning and memory tests. Three modalities were used to measure their learning and memory: delayed response task, color discrimination and spatial discrimination. The delayed response task tests monkeys to remember which well contained food after a certain time of delay. The correct rates in the test of color discrimination, spatial discrimination and each delayed time in the training phase and testing phase of the delayed response task were not different between the control animals and animals exposed to sevoflurane by t-test (Fig. 2). Sevoflurane exposure was not a significant factor to determine the correct rates of animals in the delayed response task by two-way analysis of variance with the delayed time and sevoflurane exposure as the two factors in the analysis.

### Sevoflurane exposure did not reduce neuron-specific proteins in the fontal cortex and hippocampal CA3

**Figure 3:**
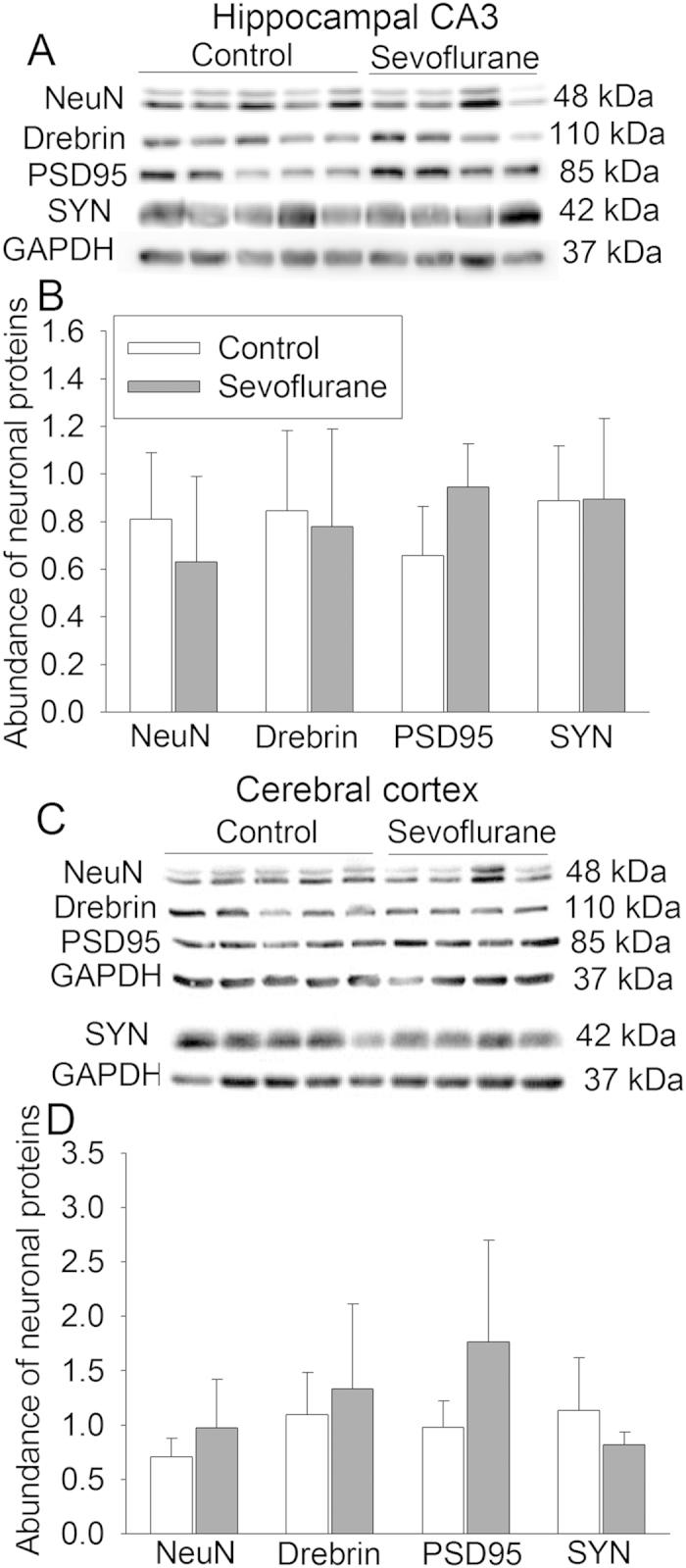
Neuron-specific protein expression after sevoflurane exposure. Six-day old male Cynomolgus monkeys were exposed to sevoflurane anesthesia for 5 h. Brain was harvested 10 months after sevoflurane exposure for Western blotting. **A**: Representative images of protein expression in the hippocampal CA3. **B**: Pooled results after normalizing the data of interesting proteins by those of glyceraldehyde 3-phosphate dehydrogenase (GAPDH) in the hippocampal CA3. **C**: Representative images of protein expression in the frontal cerebral cortex. **D**: Pooled results after normalizing the data of interesting proteins by those of GAPDH in the frontal cerebral cortex. Another Western blot was performed for the samples of the frontal cerebral cortex because synaptophysin protein band was not clearly resolved with the first blot. Results are mean ± S.D. (n = 4 – 5). PSD95: postsynaptic density protein 95, SYN: synaptophysin.

We chose to determine the expression of four neuron-specific proteins. NeuN is a nuclear protein in the neurons. Drebrin is a dendritic protein. Postsynaptic density protein 95 (PSD95) and synaptophysin are synaptic proteins^10^,^29^. The expression of PSD95 tended to be increased in the hippocampal CA3 and frontal cortex of animals exposed to sevoflurane but this increase was not statistically significant (P = 0.065 and 0.11 for CA3 and fontal cortex, respectively). Sevoflurane exposure did not affect the expression of NeuN, drebrin and synaptophysin in the hippocampus and cerebral cortex (Fig. 3).

## Discussion

Our results showed that sevoflurane anesthesia in newborn monkeys did not affect behavior, learning and memory in their childhood. Consistent with these functional data, sevoflurane anesthesia did not reduce the expression of neuron-specific proteins in the 10-month old monkeys after the learning and memory tests were completed. These findings may suggest the safety of sevoflurane anesthesia in pediatric patients.

Many studies have been performed to determine the effects of sevoflurane on developing brain in rodents. Most of these studies have shown that sevoflurane exposure increases brain cell apoptosis^20^,^22^, although no increase of cell injury after being exposed to sevoflurane at clinically relevant concentrations for a long time has also been reported in rats^23^,^30^. In addition, previous studies have shown that anesthesia with isoflurane, propofol or ketamine induces brain cell injury in newborn or fetal monkeys^11^,^12^,^13^,^31^. Cells that are injured include neurons and non-neurons and are distributed in many areas^11^,^12^,^31^,^32^. However, exposure to 1% isoflurane for 8 h or dexmedetomidine infusion for 12 h does not induce cell death in the newborn or fetal monkey brains^33^,^34^. Concentrations of isoflurane to maintain surgical level of anesthesia are about 1.5% in monkeys^35^. Dexmedetomidine is an intravenous sedative agent. Of note, the assessments of cell injury/death in these previous studies were performed in a few hours after anesthetic exposure and mostly reflected by using indices of cell apoptosis. It is not clear whether this early cell injury/death results in a reduced number of neurons. A previous study showed that exposure of 14-day old fetuses to sevoflurane reduced PSD95 and synaptophysin expression in the brain of mice when they were about one month old^36^. In our study, the expression of multiple neuron-specific proteins is not affected except for PSD95 that tended to be increased in the hippocampus and frontal cortex of monkeys exposed to sevoflurane. Since this measurement was performed with brain tissues harvested about 10 months after sevoflurane exposure, our results suggest that sevoflurane does not result in a significant change in neuronal structures in the brain. The reasons for the different findings between our study and the previous study using mice are not clear. Different species and exposure times may contribute to the different findings. Consistent with our findings from monkey study, we have shown that volatile anesthetics including sevoflurane do not injure human neuron-like cell cultures^29^.

An important question is whether the early cell injury/death after anesthetic exposure leads to cognitive or behavioral impairment. There are only one prior study using non-human primates to examine whether anesthetic exposure early in life affects cognitive functions. That study showed that a prolonged ketamine infusion (for 24 h) to neonatal monkeys induced cognitive impairment^14^. Although a previous study from the same group of investigators revealed that this ketamine exposure caused brain cell death^37^, the role of cell injury in the cognitive impairment is not known because it is not determined whether the brain cell death is just an association with cognitive impairment. A recent study showed that exposure of newborn mice to isoflurane and propofol induced significant cell injury in the brain but did not lead to learning and memory deficits^38^, suggesting that early cell injury/death after anesthetic exposure does not necessarily lead to learning and memory impairment in mice. Our study showed that exposure of neonatal monkeys to sevoflurane for 5 h did not affect their performance in multiple tasks of learning and memory. Our results also showed that sevoflurane exposure did not affect the behavior of the monkeys, an important aspect that is often not measured in previous animal studies determining anesthetic effects on the brain^14^,^36^. Our results suggest that sevoflurane anesthesia for 5 h does not cause long-lasting effects on behavioral and cognitive functions, implying the safety of sevoflurane anesthesia in humans.

Interestingly, the holding cage behavioral tests revealed that age was a significant factor for determining the frequency of stress events, hanging and environmental exploration activity. The frequency of these events or activities was more when the monkeys were 3 months old than that when they were 7 months old. These monkeys were weaned at the age of 7 months. The increased stress events and hanging activity in 3 month old monkeys during the test may be part of separation anxiety that often occurs in humans between 1 to 4 years of age^39^. The curiosity to new environment and separation anxiety may also increase environmental exploration activity in those younger monkeys.

We exposed 6-day old monkeys to sevoflurane. The brain maturity of this age of monkeys may correspond to that of 6-month old human brain. Although they are not in their maximal brain growth rates, their brain is still in the fast growth phase^31^. In addition, previous studies have shown the sensitivity of the brain at this monkey age to general anesthetics for brain cell injury/death^11^,^12^,^31^,^32^,^33^,^34^.

Sevoflurane is used to induce and maintain anesthesia in humans and is by far the most commonly used general anesthetic in children. To simulate clinical situation, we maintained sevoflurane anesthesia at surgical plane. Anesthesia for 5 h is longer than many pediatric surgeries. This length of anesthesia was chosen to provide sufficient time x dose of sevoflurane exposure to determine the effects of sevoflurane on monkey brain. In addition, studies have shown that anesthesia with isoflurane, propofol and ketamine for 5 h induces cell injury in the monkey brain^11^,^12^,^13^,^31^.

Most previous studies on the anesthetic effects on developing brain were performed in Rhesus macaque^7^,^8^,^9^,^17^,^18^. We used Cynomolgus macaque. These two species are very closely related and can reproduce viable hybrids. However, Cynomolgus macaque is smaller than Rhesus macaque (by about 10%). Different responses to the anesthetic effects have not been documented between Cynomolgus macaque and Rhesus macaque.

Our studies have limitations. First, we did not determine whether sevoflurane induces an immediate brain cell injury/death in 6-day old monkeys. We showed no reduction of neuron-specific proteins in the hippocampus and frontal cortex at 10 months after sevoflurane exposure. Measurement at this time may not reflect the cell injury in the acute phase after anesthetic exposure. Since monkeys are an extremely precious resource, we feel that it is very important to determine the long-term effects of sevoflurane anesthesia on monkey brain, especially on the behavior and cognitive functions. Second, the number of animals in our study is relatively small. The number of animals in each group in the previous studies to determine anesthetics-induced brain cell injury/death or cognitive impairment is from 3 to 6 monkeys^11^,^12^,^13^,^31^,^32^,^33^,^34^. The standard deviation of the data from delayed response task, color discrimination and spatial discrimination was about 20% or less of the mean values in our study. An experiment with 5 animals in one group and 6 animals in another group should detect 38% difference in mean values with a standard deviation at 20% and a desired power of 0.8 at an α level of 0.05 by t-test. Thus, we have sufficient numbers of animals to detect a moderate impairment in learning and memory between the two groups of animals with a good power. In addition, many different modalities testing cognition and behavior as used in this study have shown that sevoflurane anesthesia in neonatal monkey may not affect their cognition and behavior in childhood, which increases the confidence on the conclusion. Finally, we studied sevoflurane exposure only. It has been shown that nociceptive stimuli in addition to anesthetic exposure increase brain cell death and cognitive impairment in neonatal rats^40^. It will be very interesting to determine the effects of surgery plus anesthesia on behavior and cognition in non-human primates in future studies.

In summary, our data suggest that exposure of neonatal monkey to surgical plane of sevoflurane anesthesia for 5 h may not significantly affect their cognition, behavior and brain neuronal structures in childhood. This finding suggests the safety of sevoflurane anesthesia in children. Our study also suggests an age-dependent separation anxiety behavior in young monkeys.

## Materials and Methods

### Animals

This study was approved by the animal care and use committee at Sun Yat-Sen University and performed in strict accordance with the National Institutes of Health Guide for the Care and Use of Laboratory Animals (NIH publications number 80-23) revised in 2011. Efforts were made to minimize suffering of animals.

Twelve male Cynomolgus monkeys at postnatal day 6 were provided by Southern China Primates Research Center (Guangdong Landau Biotechnology Co. Ltd, Guangdong, China). They were randomly divided into two groups: sevoflurane group (n = 6) and control group (n = 6). These monkeys were kept with their mothers and other monkeys in a room of about 80 m^2^. They had free access to food and water. They were removed temporally from the group when they were subjected to behavioral, learning and memory test. The young monkeys were weaned from their mothers at the age of 7 months and then lived in a different room. All rooms were maintained at 21 °C to 24 °C with a 12-hour light-dark cycle (light from 06:00 to 18:00).

### Sevoflurane exposure

Sevoflurane exposure was performed in a way similar to that of isoflurane exposure described before^31^. A monkey was placed in a box that was gassed with 3% sevoflurane (Maruishi Pharmaceutical Co., Ltd.) in oxygen. Once his breathing became slow and regular, the monkey was removed from the box and masked with 3% sevoflurane until the end tidal sevoflurane concentrations reached 2.6 to 2.8%. The monkey was tracheally intubated and mechanically ventilated (small animal ventilator; Harvard Apparatus, Holliston, MA) to maintain end tidal CO_2_ at ~40 mmHg. Our goal was to maintain a surgical plane of anesthesia that was defined to be no body or limb movement and no more than 10% increase in heart rate in response to tail pinching. Initial sevoflurane concentration was set at 2%. If the criteria of surgical plane were not met after tail pinching, sevoflurane concentration was increased by 0.2%. The tail was pinched again 5 min later. Sevoflurane concentration will be increased by 0.2% if animal responded to the pinch. This process was repeated until the animal did not respond to the stimulus. If the animal did not respond to the initial stimulus, sevoflurane concentration was decreased by 0.1% every 5 min until the animal responded to the stimulus. Sevoflurane concentration was then increased by 0.2% and this concentration was used to maintain the anesthesia. Animals were then pinched again every 30 min and sevoflurane concentrations were adjusted as above to maintain anesthesia at surgical plane for 5 h. The end-tidal gases (Capnomac; Datex Ohmeda, Madison, WI) and peripheral blood oxygen saturation were continuously monitored during anesthesia. A tail arterial line was placed to draw blood for gas analysis at 1, 3 and 5 h after the onset of anesthesia. During the exposure, monkeys were kept warm by placing them on an electric blanket at 38 °C. A tail vein line was placed for continuous fluid and glucose infusion (5% glucose saline) at 4–8 mg/kg/h. Animals were extubated after anesthesia and then placed in a box gassed with oxygen for recovery. After recovery, they were returned to their mothers. Animals in the control group were not subjected to any treatments or separation from their mothers at this time.

### Behavioral observation

Animals were subjected to the holding cage test when they were 3 or 7 months old. During the test, a monkey was placed into a big cage in a room that did not have other animals or humans. The mother’s sound could not be heard in this room. Both the cage and room were new to the monkey. The monkey was videotaped using a high-resolution portable digital video camera fixed on a tripod for 2 h at the age of 3 months and for 1 h at the age of 7 months. This videotape started when they were placed into the cage. All monkeys were videotaped at the same time of the day (10:00 to 12:00 at the age of 3 months and 10:00 to 11:00 at the age of 7 months).

The behavioral parameters were selected according to the previous studies^26^,^27^,^28^. The frequency of stress events and environmental exploration as well as the time spent on locomotion and hanging were determined. Stress events consisted of aggressive behaviors and submissive behaviors. Aggressive behaviors included stare, threat, mouth opening threat, chase, displacement, bite, slap and grab. Submissive behaviors included lip smack, grimace, submissive presentation, moving away, scream, scream threat, crouch and fleeing. Hanging is considered an escape response^27^. The definitions of these behaviors were described in the previous studies^26^,^27^,^28^. The behavior of all monkeys in the first 15 min and last 15 min in the videotapes was analyzed blindly by one technician. The results of these two 15-min were then pooled together.

### Learning and memory test

The learning and memory of monkeys were tested using a Wisconsin General Testing Apparatus (WGTA). WGTA is often used to determine the learning and memory of non-human primates and is suitable for testing primates of all ages^41^,^42^. In our study, monkeys were subjected to delayed-response task, color discrimination and spatial discrimination test. At first, monkeys were trained to learn how to uncover the lid of a well. After that, animals were subjected to the tests in the following sequence: delayed-response task, color discrimination and then spatial discrimination test.

#### Delayed-response task

As described previously^41^, monkeys were trained to perform a delayed-response task to test their working memory in a WGTA. Briefly, monkeys did not have access to food for 4 to 6 h. They were presented with two spatially displaced wells and watched food (a piece of apple) being placed into one well. The wells were then covered with identical plaques. An opaque screen was placed between the monkey and the test wells for a specified delay. After the delay, the screen was removed to allow the monkey to choose the well for food. Two phases of delayed-response task, training phase and testing phase, were performed. Five delay lengths, 1, 5, 15, 30 and 60 s, were used in both phases. Animals were subjected to 30 trials a day, 20 s interval between trials, on the same time delay during the initial training phase. They were trained on the same time delay for at least 4 days and then moved to the next longer time delay if they achieved 70% correct rate of identifying the baited well. If this goal was not achieved, up to 6 additional training days were used for this time delay, i.e., maximally 10 days for each time delay. Total 1000 trials per monkey were performed. If a monkey had completed the training of all time delays with fewer than 1000 trials, the monkey will be trained again, 30 trials a day, on all five delays (6 trials per delay) in each day until 1000 trials were performed. The testing phase started the next day. The monkeys were subjected to 30 trials a day on all five delays (6 trials per delay) for 5 days. The correct rates for each time delay in the first 4 days during the training phase and in the 5 days during the testing phase were recorded and analyzed.

#### Color discrimination test and spatial discrimination test

Discrimination tests can be designed to change complexity to suit subjects of all ages^41^. A basic procedure of discrimination tests includes providing two distinct stimuli. One stimulus is considered correct and is reinforced with a reward (a piece of apple in this study); whereas the other is designated incorrect and is not reinforced. Trials are performed with the location of the correct stimulus randomly changed. In our study of color discrimination, red color was considered correct stimulus. Each monkey was subjected to 100 trials after they made the first correct choice. These 100 trials were performed in 2 consecutive days (50 trials per day with a 20-s interval between trials). This test is often used to test monkeys younger than 6 months or older monkeys with significant cognitive impairment. The spatial discrimination test is a more challenging paradigm. Spatial cues are needed to be identified to learn a specific physical location, such as the left or the right. In our study, left well was considered as the correct choice. Monkeys were rewarded with a piece of apple if they made the right choice. The next day after the completion of color discrimination, every monkey was tested for 100 trials in two consecutive days after he made the first correct choice. The correct rates in the 100 trials of the color and spatial discrimination tested were calculated.

### Western blotting

After the behavioral and cognitive tests were finished (the monkeys were about 10 months old), they were deeply anesthetized by sevoflurane and transcardially perfused with phosphate buffered saline at 4 °C. Their brains were harvested and kept at −70 °C.

Western blotting was performed as we described before^7^,^43^. Briefly, 30 μg proteins of the hippocampal CA3 region and frontal edge of frontal cortex were loaded per lane and separated by sodium dodecyl sulfate-polyacrylamide gel electrophoresis (SDS-PAGE). They were transferred onto nitrocellulose membranes. The membranes were blocked with 5% skimmed milk for 1 h at room temperature. The membrane was then incubated with the following primary antibodies: rabbit polyclonal anti-PSD95 (1:1000 dilution, catalog number: ab18258, Abcam), rabbit polyclonal anti-synaptophysin (1:1000 dilution, catalog number: ab14692, Abcam), rabbit polyclonal anti-drebrin (1:1000 dilution, catalog number: 5052S, Cell Signaling Technology), rabbit polyclonal anti-NeuN (1:1000 dilution, catalog number: ab177487, Abcam), and rabbit polyclonal anti-GAPDH (1:1000 dilution, catalog number: 2118S, Cell Signaling Technology) at 4 °C overnight. The membranes were then washed by tris-buffered saline with Tween 20 (three times, 10 min each). The membranes were incubated with a secondary antibody (1:2000 dilution, catalog number: 7074S, Cell Signaling Technology) for 1 h. The membranes were washed again by tris-buffered saline with Tween 20 for three times, 10 min each time, before protein bands were visualized by chemiluminescence method. Images were quantified using the software ImageJ2x. The relative expression of the interesting proteins was normalized by that of GAPDH in the same sample.

### Statistical Analysis

Results are presented in mean ± S.D. or median (range). Data were analyzed by t-test or two-way analysis of variance as appropriate. Differences were considered significant at P < 0.05. Statistical analyses were performed using SigmaStat (Systat Software, Inc., Point Richmond, CA, USA).

## Additional Information

**How to cite this article**: Zhou, L. *et al.* Neonatal exposure to sevoflurane may not cause learning and memory deficits and behavioral abnormality in the childhood of Cynomolgus monkeys. *Sci. Rep.* **5**, 11145; doi: 10.1038/srep11145 (2015).

## Figures and Tables

**Table 1:** **Physiological results of sevoflurane group.**

		**Time after the onset of anesthesia**		
**Before anesthesia**		**1** **h**	**3** **h**	**5** **h**
HR, beats/min	178 (166–195)	140 (131–155)	144 (135–152)	138 (130–158)
pH (arterial)	---	7.292 (7.278–7.369)	7.331 (7.247–7.381)	7.335 (7.274–7.395)
PaCO_2_, mmHg	---	43.9 (32.4–53.9)	45.6 (33.4–49.5)	48.5 (37.2–53.5)
PaO_2_, mmHg	---	211 (170–248)	245 (188–338)	223 (178–256)
HCO_3_^−^, mmol/L	---	21.2 (18.7–23.9)	23.8 (21.1–25.7)	24.0 (22.7–27.8)
Hb, g/dL	---	15.3 (13.9–16.3)	14.6 (13.9–15.6)	14.0 (13.3–16.7)
Glucose, mmol/L	---	4.9 (3.7–5.6)	5.6 (4.1–6.2)	5.3 (4.4–6.3)
SpO_2_, %	99.0 (98–100)	99.6 (99–100)	99.8 (99–100)	99.5 (99–100)
ETsevo, %		2.3 (2.2–2.5)	2.4 (2.2–2.6)	2.3 (2.0–2.6)

Data are presented as median (range). HR: heart rate, PaCO_2_: arterial carbon dioxide tension, PaO_2_: arterial oxygen tension, HCO_3_^–^: arterial bicarbonate radical, Hb: hemoglobin, SpO_2_: pulse oxygen saturation, ETsevo: end-tidal sevoflurane concentrations.
